# Are Poultry or Wild Birds the Main Reservoirs for Avian Influenza in Bangladesh?

**DOI:** 10.1007/s10393-017-1257-6

**Published:** 2017-06-15

**Authors:** Mohammad Mahmudul Hassan, Md. Ahasanul Hoque, Nitish Chandra Debnath, Mat Yamage, Marcel Klaassen

**Affiliations:** 1 Centre for Integrative Ecology, School of Life and Environmental Sciences, Deakin University, Geelong, Australia; 2 Faculty of Veterinary Medicine, Chittagong Veterinary and Animal Sciences University, Chittagong, Bangladesh; 3 FAO, Dhaka, Bangladesh

**Keywords:** AIV, Antibodies, Domestic birds, Resident wild birds, Migratory birds, Spillover, Spill back

## Abstract

Avian influenza viruses (AIV) are of great socioeconomic and health concern, notably in Southeast Asia where highly pathogenic strains, such as highly pathogenic avian influenza (HPAI) H5N1 and other H5 and H7 AIVs, continue to occur. Wild bird migrants are often implicated in the maintenance and spread of AIV. However, little systematic surveillance of wild birds has been conducted in Southeast Asia to evaluate whether the prevalence of AIV in wild birds is higher than in other parts of the world where HPAI outbreaks occur less frequently. Across Bangladesh, we randomly sampled a total of 3585 wild and domestic birds to assess the prevalence of AIV and antibodies against AIV and compared these with prevalence levels found in other endemic and non-endemic countries. Our study showed that both resident and migratory wild birds in Bangladesh do not have a particularly elevated AIV prevalence and AIV sero-prevalence compared to wild birds from regions in the world where H5N1 is not endemic and fewer AIV outbreaks in poultry occur. Like elsewhere, notably wild birds of the orders Anseriformes were identified as the main wild bird reservoir, although we found exceptionally high sero-prevalence in one representative of the order Passeriformes, the house crow (*Corvus splenden*s), importantly living on offal from live bird markets. This finding, together with high sero- and viral prevalence levels of AIV in domestic birds, suggests that wild birds are not at the base of the perpetuation of AIV problems in the local poultry sector, but may easily become victim to AIV spill back from poultry into some species of wild birds, potentially assisting in further spread of the virus.

**Electronic supplementary material:**

The online version of this article (doi:10.1007/s10393-017-1257-6) contains supplementary material, which is available to authorized users.

## Introduction

Globally avian influenza is one of the most potent zoonotic diseases affecting poultry, but some strains can also have the potential to affect wildlife and human health (Bahl et al. 2016; Peiris et al. 2007), with the always looming potential of a pandemic of H1N1 Spanish Flu dimensions. Outbreaks of highly pathogenic avian influenza (HPAI) H5N1 [Goose/Guangdong/1/1996 (Gs/GD)], which started in China in 1996 and rapidly spread across the globe thereafter, have reawakened fears and have thus far led to huge economic losses in the domestic poultry industry, several outbreaks in wildlife and, although far from a pandemic order, considerable loss of human life (Wikramaratna et al. 2014). In its wake, other problematic strains of avian influenza virus (AIV) have recently emerged such as H7N9 (A/Zhejiang/DTID-ZJU01/2013) in China in 2013 and South Korea in 2015 (Chowell et al. 2013; FAO 2015; Samantha et al. 2016). Moreover, in as many as nine countries in Southeast Asia, the Middle East and North Africa, HPAI H5N1 [Goose/Guangdong/1/1996 (Gs/GD)] is now considered endemic adding to its economic impact and its health risk to local wildlife and human health (Olsen et al. 2006; Peiris et al. 2007). These threats continue to demand studies that identify the causes for the emergence and spread of AIV and methods for its containment.

Wild waterbirds from the orders Anseriformes (including ducks, geese and swans) and to a lesser extend Charadriiformes (including gulls, terns, sandpipers and plovers) are recognized as the natural reservoirs of influenza A viruses (Caron et al. 2016; Nishiura et al. 2009; Vandegrift et al. 2010). Migratory representatives of these orders are thought to serve as important vectors for AIV, expanding the geographic distribution of the virus (Samantha et al. 2016; Verhagen et al. 2015; Webster et al. 1992). Low prevalence in other bird orders, such as the large order of passerine songbirds, suggests that these are primarily spillover hosts having been infected through contact with poultry or water birds (Fuller et al. 2010). However, among these some peri-domestic species, such as house sparrows (*Passer domesticus*), may still have a role in moving viruses between poultry farms (Bahl et al. 2016; Vandegrift et al. 2010) or other wild birds and farms (Prosser et al. 2013).

The outbreaks of HPAI H5N1 [Goose/Guangdong/1/1996 (Gs/GD)] in Asia and its subsequent spread in Russia, the Middle East, Europe and Africa, as well as concomitantly occurring outbreaks of the same virus in wild birds in Qinghai Lake, China (Li et al. 2011), and various places throughout Europe (Adlhoch et al. 2014; Newman et al. 2012), led to an increased focus on wild birds as reservoirs and vectors for AIV (Olsen et al. 2006). This focus intensified with the discovery that some waterbirds respond asymptomatically to certain HPAI infections (Lebarbenchon et al. 2010). The recent rapid spread of HPAI H5N8 from South Korea to Europe and North America has provided a new incentive for the study of wild birds as vectors for HPAI dispersal (Bevins et al. 2016; Verhagen et al. 2015). HPAI H5N8 is currently spreading in European countries with very recent detection in wild birds in Germany (OIE report 21470, 2016), Switzerland (OIE report 21485, 2016), Denmark (OIE report 21498, 2016) and the Netherlands (OIE report 21515, 2016).

However, various subtypes of AIV continue to also be detected in a range of domestic birds, including some HPAI strains isolated from apparently healthy domestic ducks (Kim et al. 2009). It has thus also been hypothesized that in large parts of Asia domestic ducks are an important part of the reservoir community, acting as asymptomatic carriers, remaining unaffected and, hence largely undetected, while susceptible domestic species like chickens and turkeys continue to suffer high mortality (Kim et al. 2009). Rapidly increasing demand for poultry products, poor biosecurity and the trade of poultry via live poultry markets (Gilbert et al. 2014) is thought to contribute to the spread of HPAI H5N1 and other HPAI in the most affected countries in the world (Peiris et al. 2007; Walsh et al. 2016). The often free-ranging nature of domestic duck production in SE Asian countries including China, Indonesia, Vietnam and Bangladesh also places these domestic ducks at the interface between wild aquatic birds and poultry, which may additionally provide them with an amplifying role in the ecology and spread of AIV (Cappelle et al. 2014).

Although wild birds are commonly suggested to play a key role in AIV dynamics, including a major function in the endemism of HPAI H5N1 in SE Asia, the number of studies where live wild birds have been sampled systematically since the establishment of HPAI H5N1 in 2003, has been remarkably few (Keawcharoen et al. 2011; Olsen et al. 2006). To determine the potential role of wild versus domestic birds as reservoirs for AIV in SE Asia, we studied the prevalence of AIV and antibodies against AIV (i.e. sero-prevalence) in wild and domestic birds in Bangladesh. Bangladesh is one of the countries in SE Asia that is frequently hard-hit by HPAI H5N1 outbreaks in poultry where HPAI H5N1 is now considered endemic. Our study concentrated on (1) whether wild birds in Bangladesh have a particularly high AIV prevalence and AIV sero-prevalence compared to domestic birds and to regions in the world where fewer AIV outbreaks occur in poultry and (2) whether wild birds of the orders Anseriformes and Charadriiformes can be identified as the main wild bird reservoirs as was earlier identified in a global data set. Finally, (3) we studied whether migratory birds, often considered being the major vehicle of global AIV dispersal, show higher prevalence than resident wild birds.

## Materials and Methods

**Figure 1:**
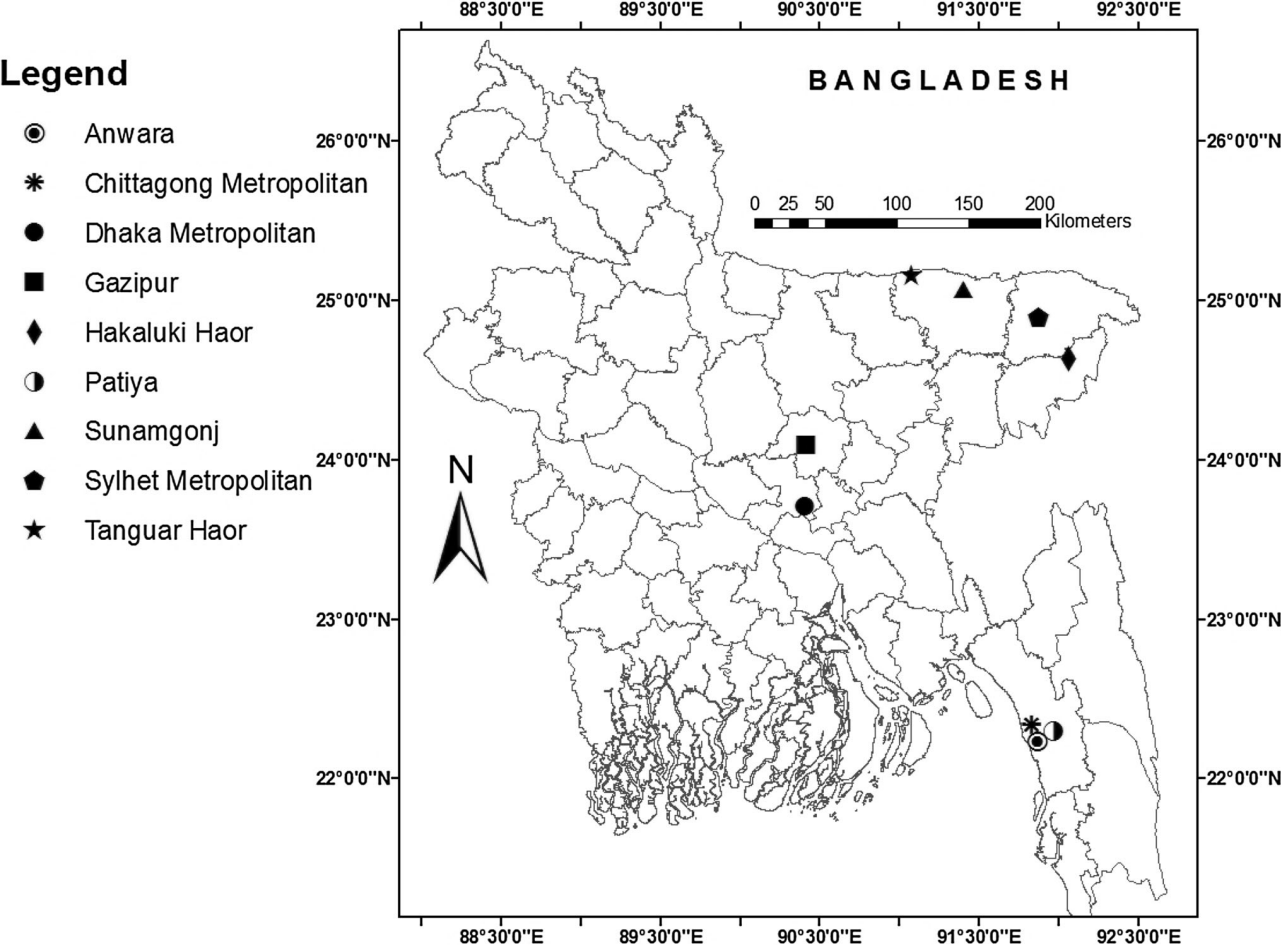
Map of Bangladesh depicting sampling locations.

We sampled a wide range of wild and domestic birds from a variety of locations across Bangladesh (Fig. 1), between May 2012 and December 2015. Although birds were sampled throughout the year, most of them were sampled during the months November through March, when also most outbreaks of HPAI occur (Biswas et al. 2014). Domestic birds were mostly sampled randomly and systematically and ranged from birds kept in commercial poultry sheds, i.e. layer and broiler (i.e. meat) chickens and chickens, ducks, quail and pigeons from live bird markets (LBMs), to birds kept on private properties in a household setting (i.e. backyard or household chickens, ducks, quail and pigeons; scientific names, order and subfamily of all bird species are provided in Supplementary Table S1), to free-ranging or range ducks, which are left unattended for most of their life after being released in wetlands at 4 weeks of age and are rounded-up for sale approximately 48 weeks later. Commercial farm chickens were sampled from 32 randomly selected farms where we targeted five samples from each farm (resulting in *n* = 159 sampled birds). Domestic or household pigeons (*n* = 13) and household chickens (*n* = 111) were randomly sampled, where we targeted one sample from each household farm and household ducks (*n* = 1232) were randomly sampled, where we targeted five samples from each household farm. Range ducks were sampled from 15 randomly selected flocks where five samples were targeted from each flock (*n* = 72) at two major wetlands, Hakaluki and Tanguar Hoar, in the vicinity of the city of Sylhet in north-eastern Bangladesh. Chickens (*n* = 27), ducks (*n* = 26), pigeons (*n* = 22), quail (*n* = 51) and spotted doves (*n* = 22) were also sampled in 20 randomly selected LBMs of Chittagong Metropolitan Area and its adjacent subdistricts of Anwara and Patiya and Sylhet and Sunamganj, Gazipur and Dhaka Metropolitan Areas, where we targeted one bird/shop. Resident wild birds (*n* = 1662), which reproduce in and are resident to Bangladesh, were sampled conveniently throughout the year, especially at roosting sites in the vicinity of LBMs, around farms in the area of Chittagong, Dhaka and Sylhet and in the wetlands of Hakaluki and Tanguar Hoar. We conveniently sampled migratory wild birds (*n* = 188), which visit wetlands in Bangladesh during the winter season (November to March) only, at the Hakaluki and Tanguar Hoar wetlands. The ultimate sample sizes varied due to mixed success in catching wild and migratory birds and convincing the general public and salesman to allow us sample their birds. All wild resident and migratory birds were caught using mist nets.

Cloacal and oro-pharyngeal swabs along with blood samples were collected from each bird except for birds from LBMs where we sampled cloacal swabs only. Swabs were taken from birds by inserting swab sticks (until faecal contamination) into the vent for cloacal swabs and oro-pharyngeal airway and wall of oro-pharynx for oro-pharyngeal swabs. Each of the cloacal and oro-pharyngeal swab samples was placed separately into a vial containing 1 ml of sterile viral transport media (Druce et al. 2012). Samples collected in the Chittagong area were stored in an insulated container with ice packs until transfer to −80°C in the laboratory at Chittagong Veterinary and Animal Sciences University (CVASU), within 2–4 h of collection. Samples collected in the areas of Dhaka and Sylhet were immediately stored in liquid nitrogen after collection.

Whole blood samples for AIV antibody prevalence analyses (0.5–3 ml, in all cases <1% of body weight) were drawn aseptically from wing veins or jugular veins and then immediately transferred to individual sterile tubes. Blood samples were subsequently allowed to clot at ambient temperature, kept refrigerated overnight, followed by centrifugation at 10,000 rpm for 30 min at 4°C to separate serum. Serum was then transferred into cryovials and preserved at −20°C (Basler et al. 1999).

Serum samples were evaluated by competitive enzyme-linked immunosorbent assay (c-ELISA) (Hoque 2011). Swabs were tested for AIV RNA using RT-PCR directed at the matrix (M) gene in an ABI Fast Real-Time PCR Machine ABI 7500 (AAHL 2014; Heine et al. 2007). For the latter, we used an Invitrogen reaction kit (Superscript^®^ iii platinon^®^ One-step Quantitative RT-PCR system—Cut No. 11732-088) and a fast cycling programme for the ABI 7500 (fast mode; thermal profile 50°C for 5 min, hold 95°C for 2 min, hold 40 cycles of: −95°C, 2 s; 60°C, 30 s).

We plotted the sampling locations on a map of Bangladesh using the spatial analyst tool of ArcGIS (ArcMap, version 10.2, Environmental Systems Research Institute, Redlands, California, USA). We used generalized linear models to analyse binomial variation in both sero- and viral prevalence across species. In the basic model, we only included species as a random factor. In a series of subsequent models of increasing complexity, we included up to two fixed factors and their interaction, with one fixed factor distinguishing between Anseriformes and non-Anseriformes and the other fixed factor distinguishing between domestic and wild birds. Using a similar procedure but for wild birds only, we also evaluated possible differences between resident and migratory birds after also distinguishing between Anseriformes and non-Anseriformes. Only species for which a minimum of ten samples was available were used in the analyses.

Our own viral prevalence in wild bird data was added to data from other researches in Bangladesh, which we extracted from the Influenza Research Database (IRD) (https://www.fludb.org/brc/influenza_surveillanceRecord_search.spg?method=ShowCleanSearch&decorator=influenza; accessed on: 12 November 2016). Bangladesh data set was compared with other data on wild birds from the Influenza Research Database distinguishing between all other countries in the world but Bangladesh where H5N1 is endemic and all countries where H5N1 is non-endemic. We distinguished between endemic and non-endemic countries using information from CDC “https://www.cdc.gov/flu/avianflu/h5n1-virus.htm”. To select the presumably wild birds only, from the IRD we selected surveillance data type “avian”, “only tested samples”, “active surveillance” and “opportunistically sampled”. Next we omitted all probable domesticated birds (i.e. all hybrid ducks and cases with species names *Anas* sp., *Anas platyrhynchos domesticus*, *Coturnix* sp., *Coturnix japonica*, *Gallus* sp., *Gallus gallus*, *Gallus gallus domesticus, Gallinago* sp., *Meleagris* sp., *Meleagris gallopavo*). All species were grouped at the level of bird order, except for species within the order Anseriformes, which were grouped at the level of subfamilies. We compared viral prevalence within the three geographic regions using a generalized linear model for binomial data with geographic region (i.e. Bangladesh, all other endemic countries and all non-endemic countries) as a fixed factor and phylogenetic grouping (i.e. order and subfamily within Anseriformes) as a random factor. Only phylogenetic groups for which at least 100 samples were available were used in the analysis. All analyses were conducted using R software (http://www.R-project.org/). For the generalized linear models procedure glmer within package lme4 was used (Bates et al. 2015). To test for the contribution of fixed factors (as well as their interactions) into the model, we used procedure lrtest within package lmtest (Zeileis and Hothorn 2002). For multiple comparisons between categories Tukey’s post hoc tests using glht in R-package multcomp were used (Hothorn et al. 2008).

## Animal Ethics

Capturing free-living birds was approved by the Bangladesh Forest Department, the Peoples Republic of Bangladesh (permit reference number: WASU/FAO/PSWMID-6/2012/58; date: 23 July 2013). Handling and sampling of birds was approved by the Chittagong Veterinary and Animal Sciences University Animal Experimentation Ethics Committee (permit ref. no. CVASU/Dir (R and E) AEEC/2015/02), Bangladesh and the Animal Ethics Committee Burwood (AECB), Deakin University (permit reference number: AEX04-2016; date: 27 July 2016), Australia. Free-living birds were released into the wild after sampling. All efforts were made to minimize animal suffering throughout our research.

## Results

**Figure 2:**
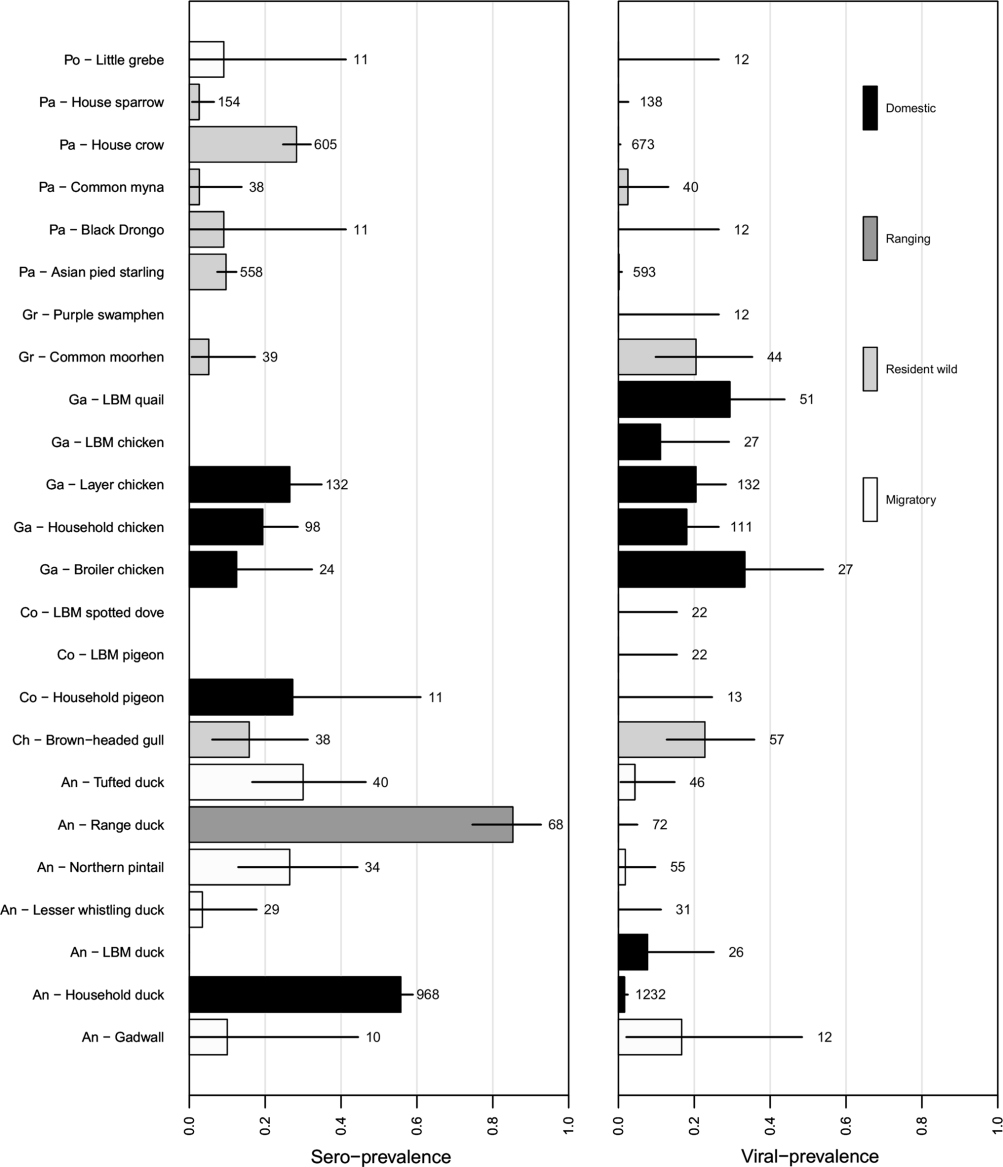
Sero prevalence (*left panel*) and viral prevalence (*right panel*) of avian influenza in domestic birds (*black bars*), semi-domestic range ducks (*dark grey bars*), resident wild birds (*light grey bars*) and migratory wild birds (*white bars*). Sample sizes and 95% confidence intervals are depicted with each bar. Only species with sample size ≥10 are depicted. Bird species along *y*-axis are arranged by order (of which first two letters are depicted) and species. For domestic birds their origin is identified as LBMs (live bird markets), household, broiler and layer chicken. For overview of all samples collected and analysed, as well as the scientific names for all species and orders (and subfamilies for Anseriformes) to which they belong, see Supplementary Table S1.

Sero-prevalence varied markedly across species ranging from 0% in broad-billed sandpiper to 85% in range duck (Fig. 2). Domestic birds (mean 43%, range 0–85%) and Anseriformes (mean 36%, range 3–85%) had a significantly higher sero-prevalence than wild birds (mean 16%, range 0–30%) and non-Anseriformes (mean 16%, range 0–31%), respectively (effect Anseriformes/non-Anseriformes: *χ* ^2^ (*df* = 1) = 6.81, *P* < 0.01; effect domestic/wild birds *χ* ^2^ (*df* = 1) = 9.84, *P* < 0.001; no significant interaction effect: *χ* ^2^ (*df* = 1) = 3.1, *P* = 0.078). Within wild birds, there was no significant difference between migratory (mean 19%, range 0–30%) and non-migratory birds (mean 17%, range 0–28%) after correcting for the effect of bird order (i.e. Anseriformes versus non-Anseriformes) [*χ* ^2^ (*df* = 1) = 0.0002, *P* = 0.98]. The major exception in these trends was the house crow (28%, 95% CI 25–32%), belonging to the order Passeriformes, which had higher sero-prevalence, similar to that observed in Anseriformes like tufted duck (30%, 95% CI 17–47%) and northern pintail (27%, 95% CI 13–44%). The trend of domestic birds having the highest sero-prevalence was even noticeable in species that are not commonly known to be reservoir species, such as pigeon (27%, 95% CI 6–61%). Still, the highest sero-prevalence was found in domestic Anseriformes, the group commonly associated with AI, especially the household duck (56%, 95% CI 53–59%) and the range duck (85%, 95% CI 75–93%) (Fig. 2).

Like sero-prevalence, viral prevalence also varied markedly, from as low as 0.2% (95% CI 0–1%) in Asian pied starling to as high as 34% (95% CI 17–54%) in broiler chicken. Interestingly, the high sero-prevalence species did not necessarily have a high viral prevalence too, with a rather low *R* ^2^ of 0.027 between sero- and viral prevalence across all species in this study. Moreover, we noted that the highest viral prevalence was found in species with low sample sizes with a concomitant large confidence interval. None of the patterns across bird groups found in sero-prevalence were mirrored in viral prevalence. Viral prevalence also tended to be higher in domestic birds and Anseriformes compared to wild birds and non-Anseriformes, respectively, but these effects were nonsignificant (effect domestic/wild birds: *χ* ^2^ (*df* = 1) = 2.3, *P* = 0.13; effects Anseriformes/non-Anseriformes: *χ* ^2^ (*df* = 1) = 0.06, *P* = 0.81; their interaction effect: *χ* ^2^ (*df* = 1) = 2.2, *P* = 0.14).

**Figure 3:**
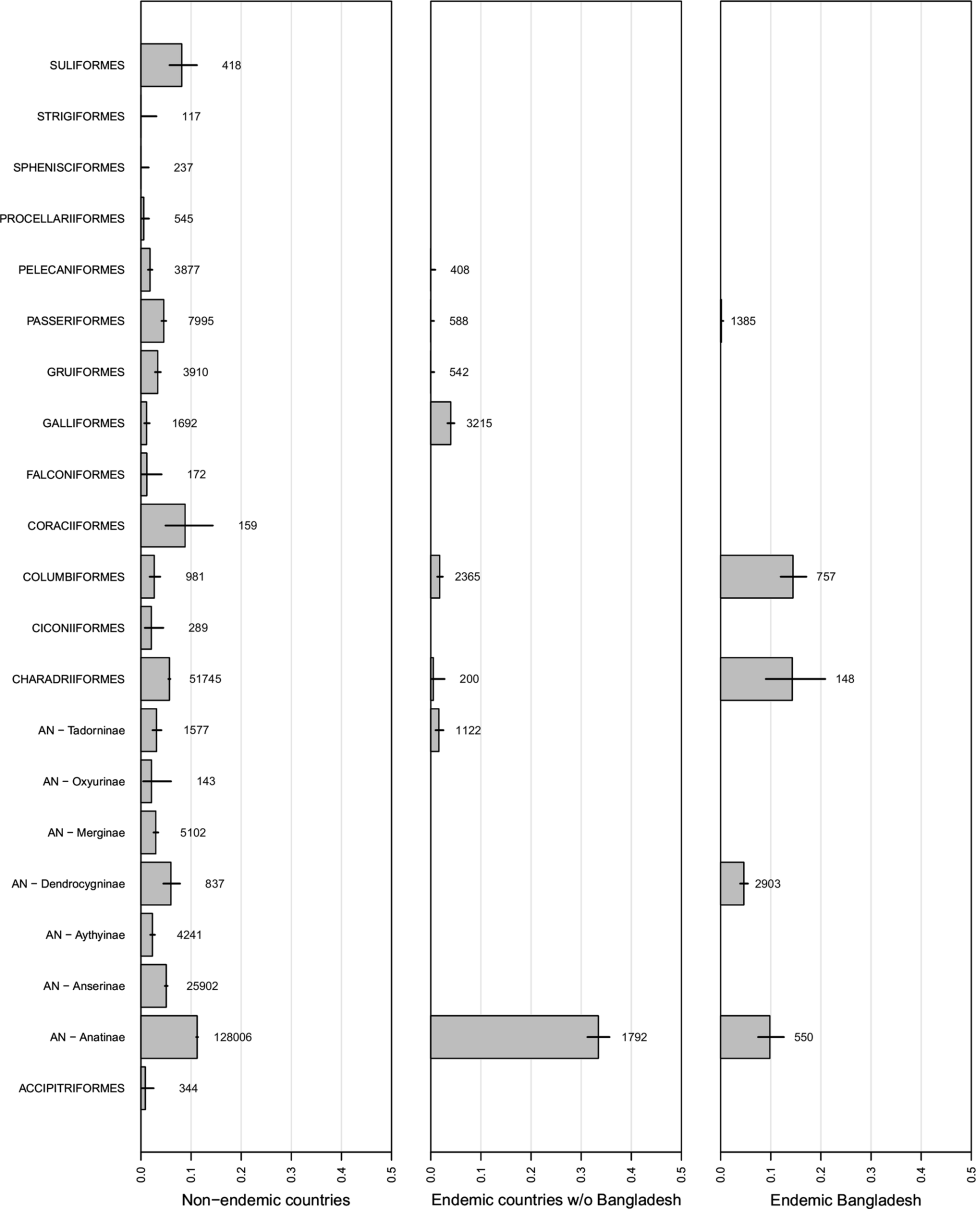
Prevalence of avian influenza virus in wild birds of different orders (and subfamilies within the order of Anseriformes) across three geographic regions: Bangladesh (*right panel*), countries where H5N1 is endemic without Bangladesh (*middle panel*) and countries where H5N1 is not endemic (*left panel*). Sample sizes and 95% confidence intervals are depicted with each *bar*. Only phylogenetic groups with sample size ≥100 are depicted.

Comparing viral prevalence found among the various orders of wild birds (and subfamilies within Anseriformes) in Bangladesh, other H5N1 endemic countries and countries where H5N1 is not endemic revealed that the pattern of prevalence varied significantly across these three geographic regions [*χ* ^2^(*df* = 2) = 257, *P* < 0.001]. AIV prevalence was significantly different between all groups (Tukey’s post hoc, *P* < 0.001), with the lowest prevalence found in non-endemic countries, and followed by Bangladesh and all other H5N1 endemic countries (Fig. 3). All endemic countries without Bangladesh particularly stood out because of a high viral prevalence in Anatinae (dabbling ducks), whereas Bangladesh itself had particularly high levels in Columbiformes (pigeons and doves) and Charadriiformes (gulls, terns and waders) (Fig. 3).

## Discussion

Generally, our data for Bangladesh suggest very high prevalence of AIV and AIV antibodies in some domestic bird groups and somewhat elevated values in wild birds when compared with data from wild birds in areas where HPAI H5N1 is not endemic. For Bangladesh, considering the need to avoid AIV infections in domestic birds for poultry and human health concerns, it is remarkable that AIV antibody prevalence among domestic birds was higher than in wild birds. Likewise, considering that wild birds are the reputed reservoir of AIV, it is also remarkable that AIV prevalence among wild and domestic birds was indistinguishable within Bangladesh. Our data therewith suggest that domestic birds may be an important part of the AIV reservoir in Bangladesh, potentially exceeding the role of wild birds.

It should be noted that we here collected and analysed data on AIV prevalence and AIV antibody prevalence and no data specifically related to HPAIV prevalence and HPAIV antibody prevalence. While there is likely exchange of virus between domestic and wild birds and different species of wild birds, and findings on AIV dynamics can be extrapolated to HPAIV dynamics to some extent, there might also be strain differentiation between different bird species and domestic and wild birds such that reservoir status might depend on particular viral strains; the relative reservoir status of domestic and wild birds might thus also be associated with particular strains (i.e. poultry-adapted or wild bird adapted strains).

AIV prevalence and AIV antibody prevalence appeared poorly correlated although research has suggested that serologic testing of wild birds could provide supportive data to advance our current understanding of AIV epidemiology (Brown et al. 2009). Since antibodies detected in serological assays last longer than viral shedding does in an actively infected animal, serological screening gives the advantage of a longer time window of detection. While serology can thus be advantageously used to assess exposure, AIV surveillance may remain of importance for assessing risk since some species may have high exposure rates and sero-prevalence, but may shed relatively little infectious virus. Moreover, not all infected birds may mount an apparent immune response (Brown et al. 2009) and this might potentially vary across species and AIV strains. Also the duration of the detectable immune response in infected birds might potentially vary across birds. Still, antibodies can be detected in blood serum for an extended period of time (i.e. in the order of months) (Curran et al. 2015; Fereidouni et al. 2010; Hoque et al. 2015; Hoye et al. 2011) and thus much longer than the few days that an AIV infection lasts. This has great repercussions for the temporal resolution requirements of the data, serological screening being more forgiving of irregular temporal spread in data than virological screening, where peaks and troughs in infection dynamics in a population can easily be missed. The low correlation (*R* ^2^ = 0.027) observed between the sero- and viral prevalence of the species sampled in Bangladesh is probably reminiscent of irregular temporal resolution in sampling. Thus, despite the above-mentioned and several other caveats on the suitability of serologic studies in elucidating AIV epidemiology (Hoye et al. 2010), serological screening possibly provides a better proxy for overall variations in susceptibility and exposure to AIV across species and regions than AIV screening, unless the AIV surveillance has been conducted systematically and at a high temporal resolution.

Overall, the wild bird species that we sampled in Bangladesh showed typical AIV prevalence and AIV sero-prevalence with Anseriformes having higher sero-prevalence than non-Anseriformes. The major exception was the house crow. House crows are omnivores often found scavenging for food wherever garbage is dumped, especially in heavily urbanized areas in Bangladesh (Koul and Sahi 2013; Shanbhag et al. 2012). All captures of house crows were made on garbage dumps in close proximity to LBMs where abundant poultry offal was present. Already in 2011, Bangladeshi house crows were reported infected by HPAI H5N1 (Khan et al. 2014). Also in other countries in Asia HPAI H5N1 viruses have been isolated from (dead) crow species, such as large-billed crows (*Corvus macrorhynchos*) in Japan in 2004 (Tanimura et al. 2006) and large-billed crow and house crow in Hong Kong in the period 2006–2007 (Ellis et al. 2009). Whereas researchers (Khan et al. 2014) considered the possibility of horizontal transmission of the virus between infected crows and poultry in LBMs in 2011, recent reports on similar cases involving house crows in Bangladesh suggest spill back from LBMs to house crows (OIE report 19727, 2016). In the face of the relatively high AIV prevalence and AIV antibody prevalence among LBMs and other poultry that we sampled, the most parsimonious explanation for the high AIV sero-prevalence observed in house crows is AIV spill back from LBMs poultry, the likely route of transmission of AIV being via contaminated poultry-offal ingestion by crows.

The threat of AIV to poultry, wildlife and human health has led to a large number of analyses attempting to identify the main risk factors explaining the potential for HPAI outbreaks in poultry. A review based on 47 published articles performed by Gilbert and Pfeiffer (2012) identified high correlations between the risk of HPAI outbreaks and domestic waterfowl presence, several anthropogenic variables (e.g. human population density and distance to roads) and indicators of open water availability, the latter often being a prerequisite for domestic waterfowl production. Although Gilbert and Pfeiffer (2012) noted that several studies considered wild birds as a potential risk factor for the emergence of HPAI in domestic birds, reliable data on the spatio-temporal distribution of wild birds were generally lacking. Instead, researchers found suggestive spatio-temporal associations between the locations of farms and habitats or migratory flyways of waterbirds, and the timing of these migrations and H5N1 outbreaks (Gilbert and Pfeiffer 2012; Si et al. 2013; Tian et al. 2015). It should be noted though that thus far identified risk factors such as habitats for wild waterbirds, water availability and domestic waterfowl presence are generally all highly correlated, making inferences on causal relationships problematic. A particularly interesting study in this respect was conducted involving the serological analyses of 24,712 wild birds and relating the results to HPAI H5N1 outbreaks in poultry (Keawcharoen et al. 2011). The final conclusion of the study was that the authors were not able to determine whether wild birds became infected because of spill back from poultry flocks or whether wild birds were the origin of outbreaks in poultry flocks, and therefore the association they found was not necessarily one of cause and effect.

The global analyses of viral prevalence data in wild birds were largely based on conveniently and unsystematically collected samples that were analysed using a variety of assays and should thus be viewed with some caution. We found AIV prevalence in wild birds in Bangladesh to be smaller than in wild birds in other countries where H5N1 is endemic but still somewhat larger to what is typically found in birds in non-endemic countries. The higher prevalence found in wild birds from H5N1 endemic countries compared to non-endemic countries could indicate a role of spill back from poultry to wild birds or that wild birds are genuinely more prone to AIV infections. Yet, given the high incidence rate of AIV outbreaks in the Bangladesh poultry sector despite the rather moderately elevated AIV prevalence values in wild birds in Bangladesh compared to non-endemic countries suggests only a limited role for wild birds in driving the AIV outbreaks in the country’s poultry. Apart from house crows, for which special considerations apply as discussed above, AIV sero-prevalence in wild birds was lower than in domestic birds, supporting the suggestion that wild birds probably play a relatively minor role in AIV outbreaks in poultry. Importantly, our data support the view that, at least in Bangladesh, domestic birds may well be a more significant reservoir for AIV than wild birds.

## Electronic supplementary material

Below is the link to the electronic supplementary material.

Supplementary material 1 (XLSX 15 kb)
